# The Merry Heart

**Published:** 1914-01

**Authors:** 


The Merry Heart.—The beneficial effect of keeping up the spirits of a patient who is wont to become depressed is well known. In fact, "to exhilarate the heart," says Burton, "has been the practice of every age and country as the best means of preserving life." Regarded as a psychological state, laughter has been thought to be merely the expression of the overflow of nervous energy, but this shows itself in many other ways than in visible mirth. According to Dr. W. McDougall, of Oxford, who read a thoughtful paper upon the subject, laughter is nature's protection against the depressing effect upon the system of our sympathetic tendencies. It may seem very ill-timed, but most of us manifest a desire to laugh when we see a person fall down, for instance. Our kindlier feelings, however, soon prevail and, controlling our risorial muscles as best we can, we hurry to the spot to render assistance. Probably, if we did not give vent to our first impulse, the "useless minor sympathetic pains," referred to by Dr. McDougall, would become too much for us, and the length of our faces would daily increase. Medical men ought, therefore, to be the cheeriest of all people and the most readily moved to laughter. But it is because of the changed direction of mental outlook induced by cheerfulness and mirth which interact powerfully even upon physical conditions that a "merry heart doeth good like medicine." The old saying of Marsilius Ficinus—"for without mirth physic is of no force"—may be heartily commended to the notice of all medical practitioners as well as psychologists.—*Medical Press and Circular.*

---

THAT INSANITY AMONG THE NEGRO POPULATION is increasing steadily is the declaration made in the annual report of Dr. William F. Drewry, superintendent of the Virginia Central State Hospital. This alarming gain of mental deficiency Dr. Drewry ascribes to congenital disease and the bad habits of parents. One of the most remarkable features of the report is the statement that 235 persons were discharged as cured of their mental infirmity, a percentage of cure almost abnormally high. Ten deaths were from pellagra, the victims having contracted the disease before entering the hospital.—*Cincinnati Lancet-Clinic.*

---

"What caused the coolness between you and that young doctor? I thought you were engaged."

"His writing is rather illegible. He sent me a note calling for 10,000 kisses."

"Well?"

"I thought it was a prescription, and took it to the druggist to be filled."—*Washington Herald.*

---

In edema of the lungs of cardiac origin a small dose of morphine often does more good than all the stimulants. It may be the only treatment needed.—*American Journal of Surgery.*

